# Characterization of canavanine-resistance of *cat1* and *vhc1* deletions and a dominant *any1* mutation in fission yeast

**DOI:** 10.1371/journal.pone.0269276

**Published:** 2022-05-31

**Authors:** Anissia Ait Saada, Alex B. Costa, Kirill S. Lobachev

**Affiliations:** School of Biological Sciences and Institute for Bioengineering and Bioscience, Georgia Institute of Technology, Atlanta, Georgia, United States of America; University of Cambridge, UNITED KINGDOM

## Abstract

Positive and counter-selectable markers have been successfully integrated as a part of numerous genetic assays in many model organisms. In this study, we investigate the mechanism of resistance to arginine analog canavanine and its applicability for genetic selection in *Schizosaccharomyces pombe*. Deletion of both the arginine permease gene *cat1* and *SPBC18H10*.*16/vhc1* (formerly mistakenly called *can1*) provides strong drug resistance, while the single *SPBC18H10*.*16/vhc1* deletion does not have an impact on canavanine resistance. Surprisingly, the widely used *can1-1* allele does not encode for a defective arginine permease but rather corresponds to the *any1-523C>T* allele. The strong canavanine-resistance conferred by this allele arises from an inability to deposit basic amino acid transporters on the cellular membrane. *any1-523C>T* leads to reduced post-translational modifications of Any1 regulated by the Tor2 kinase. We also demonstrate that *any1-523C>T* is a dominate allele. Our results uncover the mechanisms of canavanine-resistance in fission yeast and open the opportunity of using *cat1*, *vhc1* and *any1* mutant alleles in genetic assays.

## Introduction

Counter-selectable markers are widely used as genetic tools to isolate cells that experienced a mutation leading to the loss/inactivation of the marker. Counter selection in *S*. *pombe* relies mostly on the use 5-FOA (5-fluoroorotic acid) and, to a lesser extent, FudR (5-fluoro-2’-deoxyuridine) to select for mutations in the *ura4* and *TK* (herpes virus thymidine kinase) genes, respectively. In budding yeast, counterselection on canavanine, a toxic arginine analog, has been successfully integrated as a part of numerous genetic assays (e.g. [1–3]. This approach takes advantage of the *CAN1* gene that encodes for an arginine permease and confers resistance to canavanine upon its loss of function. In contrast, canavanine resistance is not part of the repertoire of markers in *S*. *pombe*, although arginine permeases and mutants conferring canavanine resistance are known. So far, counterselection on canavanine has been limited to plasmid shuffling using the *S*. *cerevisiae CAN1* gene to complement the *S*. *pombe can1-1* mutation [4, 5].

Canavanine cytotoxicity is due to its structural similarity with arginine. Canavanine is a substrate for tRNA arginyl synthase and is incorporated into nascent proteins instead of arginine. The resulting proteins harbor an altered structure and function, which leads to cells death. Therefore, in cells that do not discriminate between arginine and its toxic analog, canavanine resistance is strictly correlated with a defect in arginine uptake. The uptake of arginine, as well as other amino acids and nucleobases, is tightly regulated by the localization and expression of their cognate transporters. This regulation is sensitive to the nutritional environment and involves different signaling pathways like Tsc/Rheb in fission yeast and target of rapamycin (TOR) in both fission and budding yeasts [6–9].

In *S*. *cerevisiae*, arginine can be transported by three amino acid permeases: Can1, Gap1, and Alp1, with Can1 being the main transporter [10]. In *S*. *pombe*, several arginine permeases have been identified: Cat1 (ScGap1 homolog), Aat1 and SPBPB2B2.01 [6, 11], with Cat1 being the main arginine permease [6]. This is surprising since a widely used allele, *can1-1*, that confers strong resistance to canavanine was thought to correspond to a mutation in the gene formerly called *can1* (*SPBC18H10*.*16*) [12]. While little was known about the identity of *can1-1*, control of amino acid uptake via the Cat1 permease is well studied in fission yeast. Cellular localization of Cat1 defines the rate of arginine/canavanine uptake, and the Tsc1-Tsc2 complex is required for proper Cat1 distribution on the plasma membrane [6]. Accordingly, cells defective for the Tsc/Rheb pathway are canavanine resistant and show an intracellular mislocalizaton of Cat1. Delocalization of Cat1 from the plasma membrane occurs through endocytosis of the permease by Any1 (a β-arrestin-like protein) in cooperation with the E3-ubiquitin ligase Pub1 [13]. It has been found that over-expression of Any1 confers resistance to canavanine, while the absence of Any1 or its ubiquitination by Pub1 confers sensitivity. In addition to ubiquitination, Any1 is subject to another post-translational modification (PTM), the role and the nature of which remain unknown [13, 14].

In an attempt to make canavanine counter-selection a reliable marker in *S*. *pombe*, we undertook characterization of the *can1-1* allele. Surprisingly, we found that *SPBC18H10*.*16* deletion does not confer resistance to canavanine but rather enhances resistance in the absence of Cat1. Whole-genome sequencing of the *can1-1* strain revealed a point mutation in *any1* and no mutations in *SPBC18H10*.*16*. We found that this single mutation, *any1-523C>T*, is necessary and sufficient to reproduce the *can1-1* phenotype, *i*.*e*. strong canavanine resistance and Cat1 internalization. At the protein level, cells expressing *any1-523C>T* do not show an over-expression of Any1 but rather a strong diminution of both PTMs. These results indicate a potential role of the unknown PTM in modulating canavanine resistance. Last, we report that both Any1 PTMs are differentially regulated by the TORC1 pathway. By revealing the nature of the mutation in *can1-1*, our data add a new layer to our comprehension of the mechanisms of canavanine resistance and also the regulation of arginine uptake in *S*. *pombe*.

## Materials and methods

### Yeast strains and growth media

The *S*. *pombe* strains used in this study are listed in Table 1. Gene tagging was performed by classical molecular genetics techniques [15]. Gene deletion was performed using the *delitto perfetto* approach, initially developed in budding yeast [16]. Briefly, the pKL421 plasmid was constructed carrying a CORE cassette containing the *kanMX6* and *ura4*markers. The CORE cassette was inserted at the target locus and selected for on YES media containing G418. The CORE cassette was then replaced by transformation with an oligomer containing homology to the flanking regions of the target locus. Transformants were selected on 5-FOA containing media. Uracil auxotroph and G418 sensitive clones were selected and tested for the absence of the CORE cassette and target gene by PCR. Cat1 was tagged at the C-terminus with yeGFP and *any1* was tagged at C-terminus with six copies of HA tag using pYM25 and pYM16 (both Euroscarf) as templates for PCR, correspondingly. Oligonucleotides used in *delitto perfetto* and gene tagging are available upon request. The strain ectopically expressing the *any1-523C>T* allele was generated as follows. A ~ 1 kb region from the left arm of ChrI (I:50132–51288) was cloned into the pGEM5Zf plasmid. This region does not contain coding genes. This plasmid was used to clone the *any1-523C>T*:*kanMX4* fragment into the HpaI restriction site contained in the ChrI ~ 1 kb region. The resulting plasmid was digested with EcoRI and SacI in order to transform cells with a ~ 4.6 kb fragment containing *any1-523C>T*:*kanMX4* flanked with homology to ChrI. The resulting strains express *any1*^*+*^ from the endogenous locus and *any1-523C>T* located on the left arm of ChrI between the *plb4* and *nhe1* genes. For cell sensitivity to canavanine, serially diluted cell suspensions were spotted on EMM media supplemented with adenine, leucine and uracil and containing 5 g/L of ammonium chloride (if not otherwise stated). L-Canavanine (Sigma C9758) was added directly to the media at the indicated concentrations.

**Table 1:** Strains used in this study.

Name	Genotype	Reference
KT792	*h+ leu1-32 ura4D18 ade7Δ cat1Δ*	This study
KT794	*h- can1-1 leu1-32 ade6-M210 ura4-D18*	Gift from S. Forsbourg
KT795	*h+ can1-1 leu1-32 ade6-M216 ura4-D18*	Gift from S. Forsbourg
KT798	*h+ leu1-32 ura4-D18 ade7Δ cat1Δ can1Δ*	This study
KT1191	*h+ leu1-32 ura4-D18*	This study
KT1192	*h- can1-1*	Gift from S. Kearsey
KT1209	*h- any1*::*CORE (kanMX-ura4) in can1-1 strain*	This study
KT1241	*h+ leu1-32 ura4-D18 any1*::*CORE (kanMX-ura4)*	This study
KT1330	*h- any1*^*+*^ *can1-1 strain*	This study
KT1345	*h+ leu1-32 ura4-D18 cat1*:*GFP*:*hphMX*	This study
KT1348	*h+ can1-1 leu1-32 ade6-M216 ura4-D18 cat1*:*GFP*:*hphMX*	This study
KT1352	*h+ leu1-32 ura4-D18 any1-523C>T cat1*:*GFP*:*hphMX*	This study
KT1373	*h+ leu1-32 ura4-D18 any1*::*CORE (kanMX-ura4) cat1*:*GFP*:*Hygro*	This study
KT1391	*h+ leu1-32 ura4-D18 any1*:*HA*:*NAT*	This study
KT1394	*h+ can1-1 leu1-32 ade6-M216 ura4-D18 any1*:*HA*:*natMX*	This study
KT1397	*h+ leu1-32 ura4-D18 any1-523C>T*:*HA*:*natMX*	This study
KT1403	*h+ leu1-32 ura4-D18 any1-523C>T*:*kanMX*	This study
KT1414	*h+ leu1-32 ura4-D18 SPBC18H10*.*16*::*CORE*	This study
KT1426-1427	*h+ tor2-L2048S*:*kanMX4 (tor2-287 allele) any1*:*HA*:*natMX leu1-32 ura4-D18*	This study
KT1570	*h+ leu1-32 ura4-D18 ChrI50660*:*any1-523C>T*:*kanMX4*	This study

### Genome-wide sequencing

Two *can1-1* strains were obtained from two independent labs and used for sequencing. Whole- genome sequencing was performed by Psomagen (Rockville, MD) using Illumina TruSeq DNA Nano 350 bp library prep and an Illumina NovaSeq 6000. Reads were aligned to the *S*. *Pombe* reference genome [17] using the Burrows-Wheeler Aligner [18], Picard (http://broadinstitute.github.io/picard), SAMtools [19], and Genome Analysis Toolkit v3.8 [20]. Variants were called against the reference genome using Mutect2 [20] and referenced against genes known to affect canavanine sensitivity [21]. Sequencing data were deposited in the NCBI database with SRA SRP373038 and URL: https://trace.ncbi.nlm.nih.gov/Traces/sra/?study=SRP373038

### Whole protein extraction and western blot

Exponentially growing cells inoculated in YES were arrested with 0.1% sodium azide. 1x10^8^ cells were collected to perform the following protein extraction. The cell pellet was suspended and washed in 1 ml stop buffer (50 mM NaF, 10 mM NaN3 in PBS 1X) and then washed in 1 ml of 20% trichloroacetic acid (TCA). Pelleted cells were resuspended in 200 μl 20% TCA and glass beads (Sigma G8772) were added to each tube. The cell walls were mechanically broken using a FastPrep-24 bead beating homogenizer (MP-Biomedicals) with the following program: 6000 rpm, 3 rounds of 30 sec ON with a one-minute interval between each round on ice. 400 μl 5% TCA was added to the cell lysate and cell lysate was recovered without the beads in a new tube. The cell lysate was spun at 13k rpm for 5 min and the pellet was resuspended in 200 μl TCA buffer (1x SDS loading buffer, 0.2M Tris-HCl pH 8). The samples were denatured by boiling at 95°C for 5 min followed by a brief centrifugation prior to western blot analysis. Proteins were resolved by 4–20% SDS-PAGE gel and then transferred onto a PVDF membrane. The membrane was probed with anti-HA (1:2000, Sigma H6908) or anti-H3 (1:5000, Novus NB500-171) antibodies and the proteins were revealed by chemiluminescence with horseradish-peroxidase-conjugated sheep anti-rabbit IgG (cytiva).

### Live cell imaging and fluorescence analysis

Cells were inoculated in filtered EMM-NH_4_Cl medium supplemented with adenine, leucine and uracil. Cells were prepared for microscopy as described in [22]. Exponentially growing cells were washed in fresh supplemented EMM media and a 2 μl drop was deposited on the well of a microscope slide (Thermo Scientific, ER-201B-CE24) covered with a layer of 1.4% agarose in filtered EMM. Images were acquired with a Zeiss LSM 700A confocal microscope. Twenty-four cells from two independent cultures were analyzed. Green fluorescence protein (GFP) signal distribution was analyzed in two ways using Image J. First, three lines were drawn across the top of the indicated cell and a profile plot was generated for each line. Second, the relative intensity of GFP signal between the cell surface and the cytoplasm was determined. For that, GFP intensity was measured by drawing a line at the cell surface and three lines in the cytoplasm to minimize the non-homogeneity of the GFP signal in the cytoplasm. The relative intensity of GFP signal was calculated by dividing the intensity at the cell surface by the average intensity in the cytoplasm.

## Results

### SPBC18H10.16, formerly called Can1, plays a minor role in arginine/canavanine uptake

**Fig 1:**
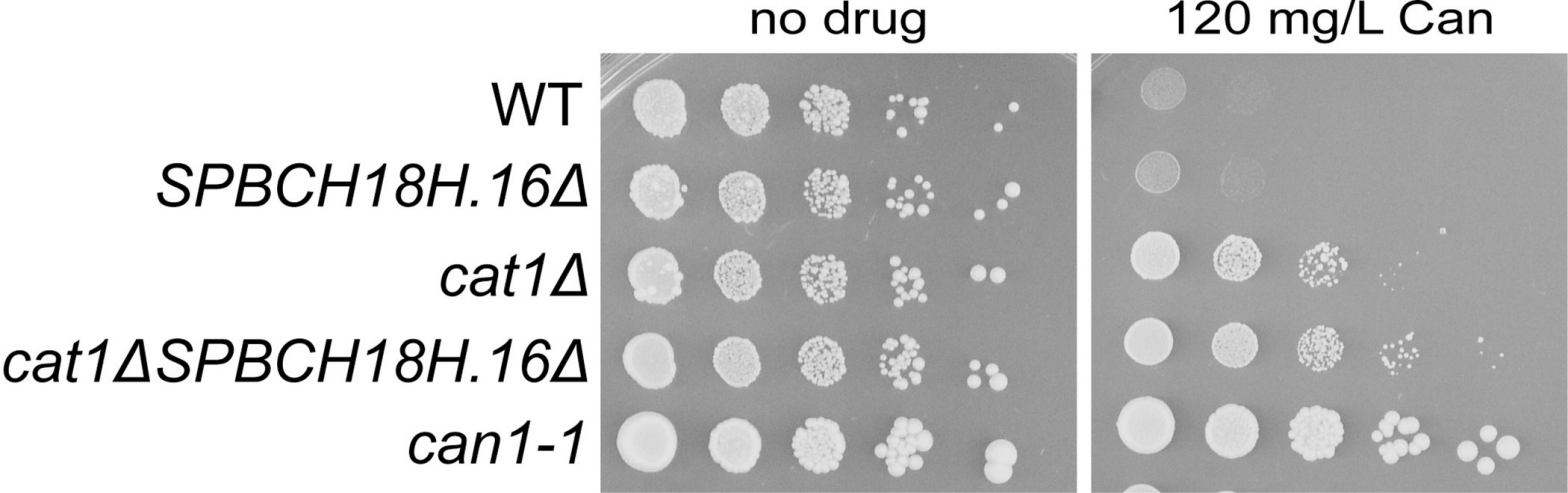
Canavanine resistance in *cat1* and *SPBC18H10*.*16* mutants. Serial 10-fold dilutions of indicated strains spotted on synthetic media (EMM) lacking arginine and containing 5 g/L of ammonium chloride in the absence or presence of canavanine (Can). The medium was supplemented with adenine, leucine and uracil. Strains used: KT1191, KT1414, KT792, KT798, KT795.

In fission yeast, expression of the *can1-1* allele and deletion of *cat1* are known to confer resistance to canavanine. The nature of the *can1-1* allele remained unknown since its discovery in 1977 [23] but, as its name indicates, *can1-1* was thought to correspond to a mutation in the *can1* gene previously assumed to be *SPBC18H10*.*16*. The allele was, indeed, referred to as defective *can1* gene in literature. However, the *SPBC18H10*.*16* gene is not used as a counter-selectable marker in *S*. *pombe*. We reasoned that if *can1-1* encodes for a defective Can1 arginine permease, deletion of *SPBC18H10*.*16* should confer resistance to canavanine to the same extent as *can1-1*. We found that deletion of *SPBC18H10*.*16* does not provide substantial resistance to canavanine compared to *cat1* deletion or *can1-1* strains (Fig 1).

Although deletion of *SPBC18H10*.*16* slightly enhances the canavanine resistance of the *cat1* mutant, the fact remains that the *can1-1* strain displays the strongest canavanine resistance. These results suggest that SPBC18H10.16 plays a minor role in arginine and canavanine uptake compared to Cat1. Importantly, these results indicate that the *can1-1* phenotype does not flow from a mutation in the *SPBC18H10*.*16* gene.

### The *can1-1* allele corresponds to a mutation in the *any1* gene

Chromosome mapping of *can1-1* showed that the corresponding gene is located on chromosome II and is linked to *tps13* (a centromere-linked marker), which is 35 map units distant [12]. Confusingly, the *SPBC18H10*.*16* gene, formerly and mistakenly named *can1*, resides around this location but is not involved in canavanine resistance (Fig 1). A simple but pertinent hypothesis explaining the *can1-1* phenotype is the involvement of another gene located in close proximity to *can1*. In order to determine the genetic basis of canavanine resistance attributed to the *can1-1* allele, we performed whole-genome sequencing of two strains from Dr. S. Forsburg’s laboratory FY254 (h- *can1-1 leu1-32 ade6-M210 ura4-D18)* and FY261 (h+ *can1-1 leu1-32 ade6-M216 ura4-D18*). During the analysis of sequencing data, we focused our attention on mutations (i) located around the deduced *can1-1* location and/or (ii) present in genes involved in canavanine resistance. One particular mutation stood out because it presented both criteria. Indeed, the *can1-1* strain showed a point mutation (523C>T) in the *any1* gene which (i) is located only 8.4 kb away from *SPBC18H10*.*16* and (ii) encodes for an arrestin-related endocytic adaptor involved in the regulation of amino acid transporters like Cat1 and Aat1. Knock out of *any1* confers an extreme sensitivity to canavanine, whereas its overexpression confers resistance to canavanine [13, 14].

**Fig 2:**
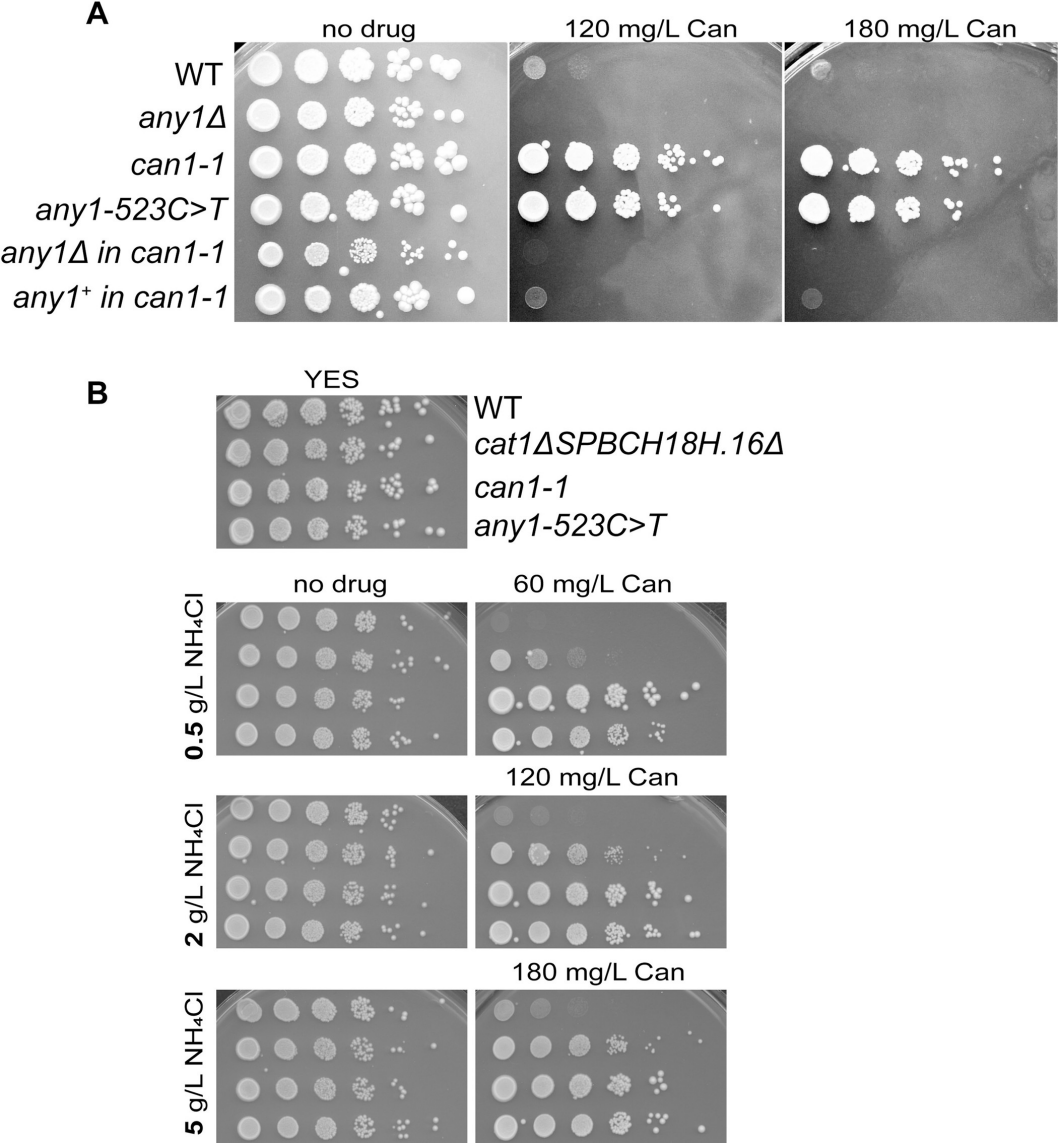
A point mutation in *any1* is responsible for canavanine resistance in the *can1-1* strain. **A.** Serial 10-fold dilutions of indicated strains spotted on synthetic media lacking arginine and containing 5 g/L of ammonium chloride in the absence or presence of canavanine. **B.** Serial 10-fold dilutions of indicated strains spotted on YES or synthetic medium lacking arginine and containing the indicated amount of ammonium chloride and canavanine (Can). Strains used in A: KT1191, KT1241, KT794, KT1403, KT1209, KT1330. In B: KT1191, KT798, KT795, KT1403.

To verify that this mutation is sufficient to recapitulate the canavanine resistance observed in the *can1-1* strain, we recreated *any1-523C>T* in our strain background and deleted *any1* in the *can1-1* strain. The 523*C>T* substitution generates a restriction site for the HpyCH4IV enzyme, which facilitates tracking of the mutation. We observed that cells expressing *any1-523C>T* show the same resistance to canavanine as the original *can1-1* strain (Fig 2A). In addition, the deletion of *any1* in *can1-1* not only suppresses canavanine resistance but also exacerbates sensitivity to the drug like observed in the WT strain lacking *any1*. Last, the replacement of *any1* gene in the *can1-1* strain with the WT *any1* sequence suppresses canavanine resistance in this strain and shows the same sensitivity as the WT strain (Fig 2A). These results show that canavanine resistance in the *can1-1* strain is not due to a defective arginine permease, like so far assumed, but rather due to a gain of function mutation in the regulator of arginine transporters, Any1. Although we found that the mutation responsible for canavanine resistance in the strain isolated by Kohli et al. [23] resides in the *any1* gene, we will keep referring to the strain as *can1-1* to distinguish it from the strain harboring *any1-523C>T* that we recreated in our strain background.

Fantes and Creanor noticed that nitrogen source impacts growth of *can1-1* in the presence of canavanine. The *can1-1* strain presented a better resistance to the drug when cells were grown on ammonium versus glutamate (two nitrogen sources used in *S*. *pombe* media). It is also known that nitrogen starvation leads to an abundance of amino acid transporters at the plasma membrane, which ensures a greater nutrient uptake [7, 13]. Therefore, canavanine sensitivity increases upon nitrogen stress because of a greater uptake of the drug. We tested if the canavanine resistance of the *can1-1* strain is impacted by the amount of available nitrogen in the media. We used 0.5, 2 and 5 g/L of ammonium chloride, the highest concentration being the standard concentration used in *S*. *pombe* media. Since decreasing the amount of nitrogen leads to a higher canavanine uptake, we lowered the concentrations of the drug in media containing 0.5 and 2 g/L of ammonium chloride. Expectedly, we observed that the extent of canavanine sensitivity in the WT strain is greater in the presence of limiting amounts of ammonium (Fig 2B). Also, the canavanine resistance of cells lacking both SPBC18H10.16 and Cat1 is lessened by limiting nitrogen availability. Interestingly, decreasing the amount of nitrogen did not have a drastic impact on canavanine resistance in the *can1-1* strain as well as *any1-523C>T* (Fig 2B). This suggests that limiting the amount of nitrogen does not lead to an accumulation of amino acid transporters at the cell surface in cells expressing *any1-523C>T*. We noticed though that *any1-523C>T* presents a slightly slower growth compared to the *can1-1* strain when cells were grown in the presence of canavanine and low amount of nitrogen (S1 Fig). This might be explained by the differences in the genetic background of the two strains.

### *any1-523C>T* is a dominant allele in haploid strains

**Fig 3:**
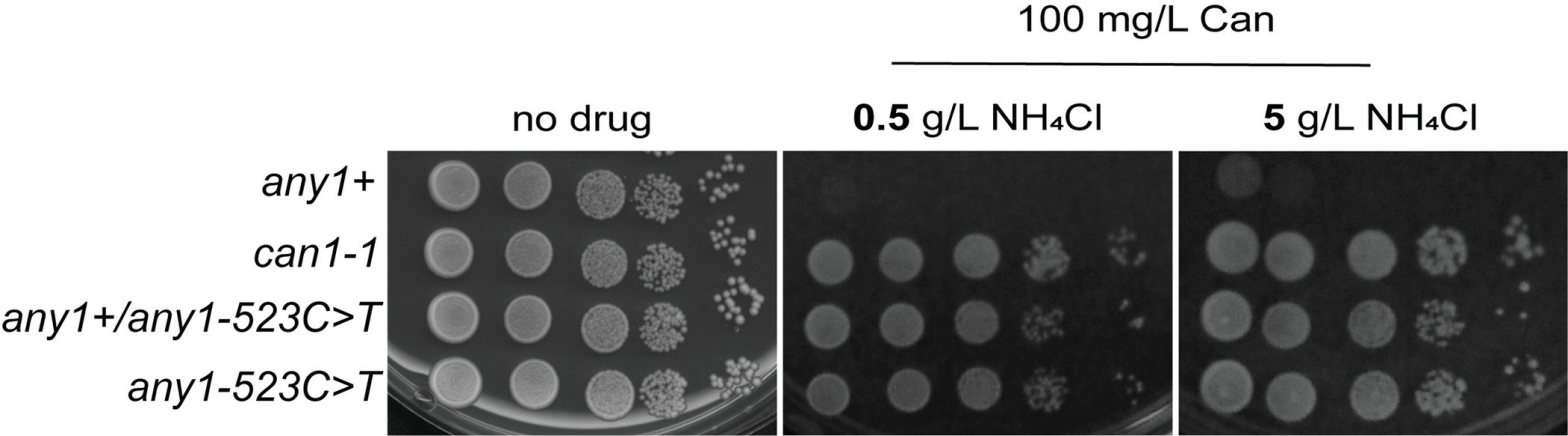
*any1-523C>T* is a dominant allele. Serial 10-fold dilutions of indicated strains spotted on synthetic media lacking arginine and containing the indicated amount of ammonium chloride and canavanine (Can). The strains are haploid and express either one copy of *any1* (*any1+*, *can1-1*, *any1-523C>T*) or two copies (*any1+/any1-523C>T*). The strain with two *any1* alleles contains *any1+* in its endogenous location and *any1-523C>T* inserted into the left arm of chromosome I. Strains used: KT1191, KT795, KT1570, KT1403.

The fact that deletion of *any1* leads to an acute canavanine sensitivity whereas *any1-523C>T* leads to a clear-cut resistance suggests that *any1-523C>T* is a gain-of-function mutation. In most cases, gain-of-function mutations are dominant or semi-dominant. To test whether *any1-523C>T* is dominant, we created a strain where *any1*^+^ is expressed from its endogenous locus and *any1-523C>T* is inserted in the left arm of chromosome I. Therefore, both a WT and a mutated copy of Any1 are expressed in the haploid strain. We found that the *any1*^+^/*any1-523C>T* strain shows the same capability to grow on canavanine-containing media as *can1-1* and *any1-523C>T* even under limiting amounts of nitrogen (Fig 3). This confirms that *any1-523C>T* is a dominant allele and also offers a great opportunity to use this allele as a selectable marker without the need to delete the endogenous gene.

### Expression of Any1^R175C^ changes the post-translational modification pattern

**Fig 4:**
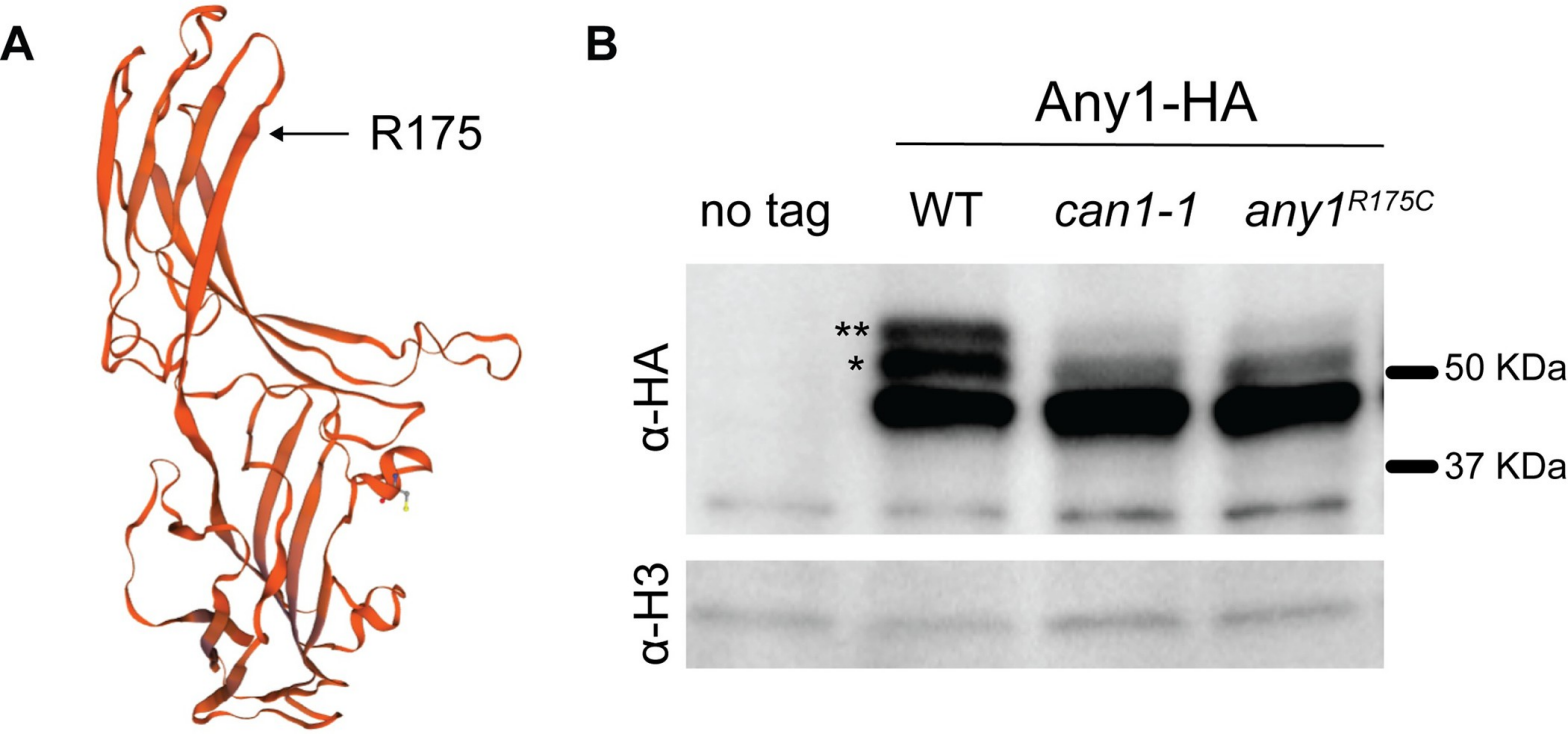
Effect of the *any1-523C>T* mutation at the protein level. **A.** Automated protein structure prediction using homology modeling (SWISSMODEL). Expression of *any1-523C>T* leads an arginine to cysteine substitution at position 175. **B.** Expression of WT and mutated Any1 (*can1-1* and Any1-R175C) analyzed by immunoblotting the endogenously tagged Any1 with anti-HA. Histone H3 is shown as a loading control. The asterisks correspond to the post-translationally modified forms of Any1, where * corresponds to an unknown PTM and ** likely corresponds to the Pub1-mediated ubiquitinated form [13]. Both PTMs are strongly diminished in cells expressing Any1^R175C^. Strains used: KT1191, KT1391, KT1394, KT1397.

To further understand the mechanism of canavanine resistance in *can1-1*, we analyzed the effect of the 523C>T mutation, which induces a change of amino acid 175 from an arginine to a cysteine. This mutation is located in the arrestin motif of the protein (Fig 4A). Nakashima et al. showed that *any1*^*+*^ overexpression confers canavanine resistance and that ubiquitination of Any1 at the K263 residue by Pub1 is required for Cat1 endocytosis [13]. Indeed, deletion of *pub1* and expression of Any1^K263R^ confer sensitivity to canavanine to the same extent as the *any1* deletion.

We tagged the endogenous *any1* gene in a WT strain and in cells expressing *any1-523C>T* (either in *can1-1* or in our strain background). As expected, on a western blot, we observed three distinct bands in the WT strain [13]: a main band and two electrophoretic mobility shifts where the upper band (*) likely corresponds to ubiquitinated Any1 [13] and the middle band (**) corresponds to an unknown PTM (Fig 4B). The protein status in *can1-1* and *any1-523C>T* was unexpected in terms of correlation with canavanine resistance. First, expression of Any1^R175C^ does not lead to a higher level of the protein that would mimic *any1*+ overexpression. Second, Any1^R175C^ does not show an increase but rather a strong decrease in its ubiquitination level. Third, the unknown PTM, the role of which is also unknown, is diminished in Any1^R175C^. This result shows that Pub1-mediated ubiquitination of Any1 is not required for canavanine resistance in cells expressing Any1^R175C^. It also suggests a potential role for the unknown PTM, loss of which may bypass the need for Any1 ubiquitination. We noted that the ratio between the three bands in WT slightly varies from one experiment to another; however, Any1^R175C^ consistently exhibits a decrease in the PTM levels.

### Expression of Any1^R175C^ leads to a massive Cat1 endocytosis

**Fig 5:**
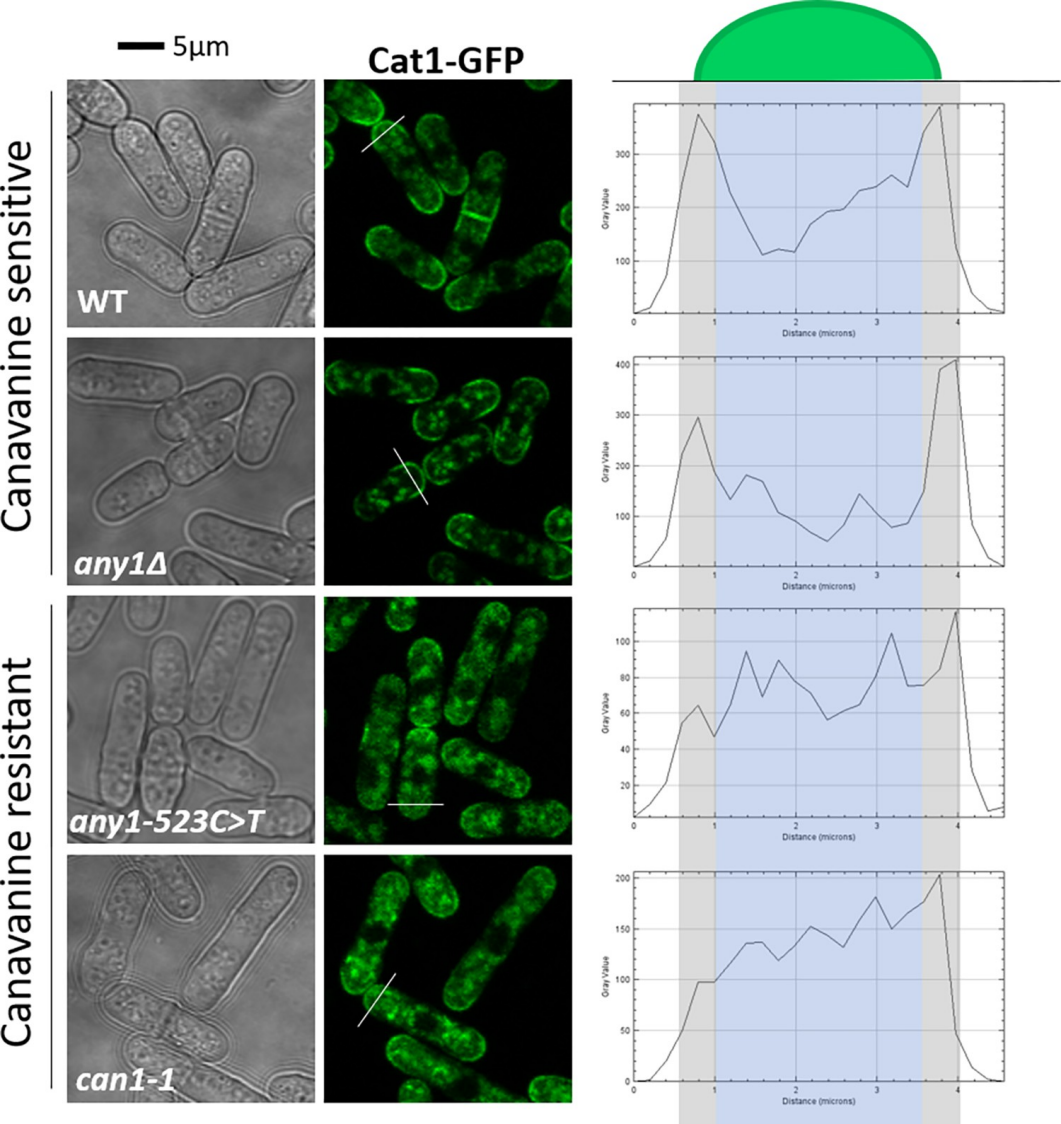
Expression of Any1-R175C leads to Cat1 internalization. Cellular localization of Cat1-GFP in indicated strains. Left panel: cells expressing Cat1-GFP observed under a fluorescence microscope. Right panel: intensity of GFP signal across the cell (white bar on the left panel). Strains used: KT1345, KT1348, KT1352, KT1373.

Any1 is involved in the regulation of amino acid transporters [14]. Under nutrient-rich conditions, it is notably responsible for storage of plasma membrane transporters, like Cat1 and Aat1, in the Golgi apparatus [13, 14]. The fact that canavanine resistance comes from the inability of the cells to import the toxic drug implies that the gain of function mutation *any1-523C>T* leads to a massive internalization of amino-acid transporters. To test this hypothesis, we tagged the native arginine permease Cat1 with GFP in order to track its cellular localization by fluorescent microscopy (Fig 5). Analysis of GFP signal across the cell (white bar on the left panel) showed two major peaks in WT and *any1Δ* at the cellular periphery. In addition, measurement of the relative intensity of the GFP signal between the cell surface and the cytoplasm shows that the ratio in WT and *any1Δ* is higher than 1 (S2 Fig). This suggests that Cat1-GFP molecules are more concentrated at the cell surface than the cytoplasm thus explaining canavanine sensitivity. Nakashima et al. showed that Cat1 accumulates at the tips of cells in response to nitrogen starvation [13]. It is important to note that the results in Fig 5 depict Cat1 localization when cells were grown overnight with a normal amount of nitrogen (EMM + 5 g/L ammonium chloride). Most likely, nitrogen is consumed in overnight cultures and requires Cat1 relocalization to the membrane. However, we found that in *any1-523C>T* and the original *can1-1* strains Cat1-GFP localization is mostly intracellular (Fig 5) and the relative intensity of the GFP signal between the cell surface and the cytoplasm shows a ratio lower than in WT and *any1Δ* (S2 Fig). This explains why the strains are very resistant to canavanine: the transporters responsible for canavanine import are excluded from the plasma membrane.

### Any1 PTMs are differentially regulated by the TORC1 pathway

**Fig 6:**
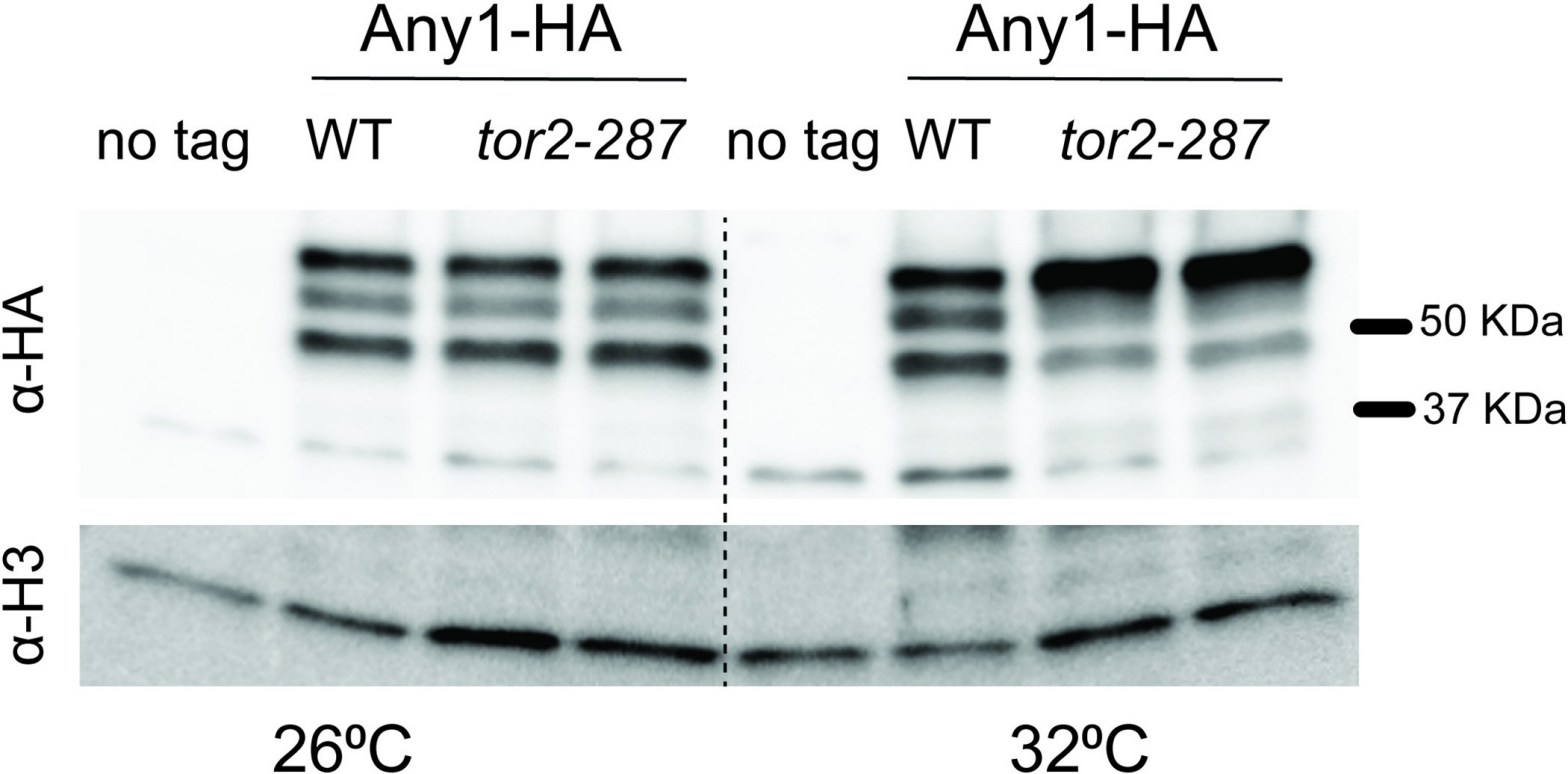
Any1 PTMs are regulated by the TORC1 pathway. Expression of Any1 in cells expressing WT *tor2* or the thermosensitive allele *tor2-287* (Tor2-L2048S). Two independent *tor2-287* clones were analyzed. The cells were grown to exponential phase and then incubated at permissive (26⁰C) or restrictive (32⁰C) temperatures. Any1 was probed by immunoblotting with anti-HA. Histone H3 is shown as a loading control. Strains used: KT1345, KT1348, KT1352, KT1373.

TORC1 and TORC2 are known to regulate arginine and leucine uptake. Tor2 is a protein kinase that is part of the TORC1 complex. It has been shown that activating mutations in Tor2 render cells more sensitive to canavanine whereas inactivating mutations confer canavanine resistance [8, 24]. The kinase defective and thermosensitive *tor2* allele (*tor2-287*, [25]) and, paradoxically, *tor2*^*+*^ overexpression confer canavanine resistance and a defect in leucine uptake [8, 26]. In *S*. *cerevisiae*, the TORC1 complex stimulates Can1-mediated arginine uptake via Npr1-mediated phosphorylation of Art1 (Any1 ortholog) [7]. The fact that *tor2-287* confers canavanine resistance and phosphorylation of the Any1 homolog in budding yeast negatively regulates its function prompted us to verify the PTM status of Any1 in cells expressing *tor2-287*. The results show that Any1 exhibits the same PTM pattern in both WT and *tor2-287* at the permissive temperature (26⁰C) but not at the restrictive temperature (32⁰C) (Fig 6). Indeed, both Any1 PTMs were differentially affected in the *tor2-287* strain. The ubiquitinated form of Any1 dramatically increased whereas the unknown PTM underwent a drastic decrease. This shows the unknown Any1 PTM is positively regulated by Tor2 and suggests that its loss is linked to canavanine resistance in *any1-523C>T/can1-1*.

## Discussion

Canavanine is a valuable drug in both fundamental and clinical research. First, counterselection using canavanine has been instrumental in developing genetic assays in budding yeast (but not in fission yeast). Second, canavanine offers a great therapeutic potential in the treatment of several cancers, including breast cancer [27–29]. Therefore, understanding the mechanism of canavanine resistance in eukaryotes is important for developing new tools in model organisms and predicting success of drug treatment in medicine.

Canavanine resistance is observed in cells lacking either an arginine permease or factors involved in the positive regulation of amino acid/arginine uptake, like the Tsc1-Tsc2 complex. *can1-1* was isolated more than three decades ago and it had been assumed that the mutation resided in the *SPBC18H10*.16/*vhc1* gene, formerly called *can1*. In this paper we show that *can1-1* corresponds to a mutation in *any1*. Concomitantly to our study, Yang et al. also found that the *can1-1* mutation resides in *any1* [30]. Accordingly, deletion of *SPBC18H10*.16/*vhc1* does not confer canavanine resistance like expected when an arginine permease is deleted. However, deletion of *SPBC18H10*.16/*vhc1* and *cat1* confers substantial canavanine resistance, even under limiting amounts of nitrogen (Figs 1 and 2B). Yet, canavanine resistance of *can1-1/any1-523C>T* is more profound than the deletion of both *SPBC18H10*.16/*vhc1* and *cat1*, suggesting that one or several other amino acid transporters regulated by Any1 are involved in arginine uptake. Accordingly, the mechanism by which canavanine resistance occurs in *can1-1/any1-523C>T* is via an excessive internalization of amino acid transporters, including Cat1 (Fig 5). It has been shown that *CAN1* from budding yeast functionally complements *can1-1* in fission yeast [5]. Since *can1-1* corresponds to *any1-523C>T*, it is likely that Any1 does not control the cellular localization of the budding yeast Can1, thus explaining the complementation. It is interesting to note that *CAN1* orthologs listed in the *S*. *pombe* database (https://www.pombase.org) correspond to several genes encoding amino acid transporters like *cat1*, *aat1*, and *isp5* but not *SPBC18H10*.*16*. Indeed, the *SPBC18H10*.*16* ortholog corresponds to *VHC1* in budding yeast, a vacuolar membrane cation-chloride cotransporter.

Besides arginine and lysine (basic amino acids), Any1 is involved in leucine uptake [31]. Usually, mutants like *tsc2* that show canavanine resistance show also a defect in leucine uptake, which prevents the mutant cells from growing on synthetic media supplemented with leucine [8, 32]. The strains used in this study are auxotrophic for leucine because they carry the *leu1-32* mutation. It was, therefore, expected that the canavanine resistance of *can1-1*/*any1-523C>T* would be accompanied by a growth defect on EMM media supplemented with leucine (no drug condition on figures). However, this was not the case (Figs 1 and 2). This suggests that the mutation *any1-523C>T* specifically affects the basic amino acid transporters involved in arginine trafficking and does not limit leucine uptake.

The fact that *any1-523C>T* is a dominant mutation that elicits canavanine resistance without affecting leucine uptake, makes this allele a newly defined selectable maker that can be used in fission yeast genetics regardless of leucine auxotrophy. It should be kept in mind that cells prototrophic for basic amino-acids (arginine, lysine and histidine) should be used. In addition to positive selection using *any1-523C>T*, we propose the use of *vhc1* and *cat1* for negative selection on canavanine. This will offer the opportunity to develop canavanine-based genetic assays in fission yeast.

It is known that Tor2 regulates amino-acids transporters both positively and negatively via a complex signaling pathway. The *ts* allele *tor2-287* that confers canavanine resistance shows an increase in *cat1* expression but no apparent massive endocytosis of the permease [8]. Nakase et al. found that Tor2 activity is not required for the formation of the Any1-Pub1 complex. Here, we show that Tor2 regulates Any1 at the posttranslational level. The increase in Any1 ubiquitination in *tor2-287* suggests that Tor2 modulates the interaction between Any1 and Pub1. This constitutes the first evidence that TORC1 regulates the function of the Any1-Pub1 complex. One common feature between *tor2-287* and *can1-1*/*any1-523C>T*, besides canavanine resistance, is a decrease in the level of the unknown PTM of Any1. It is possible that the unknown PTM corresponds to a Tor2-mediated Any1 phosphorylation. However, it has been shown recently that Any1 is subjected to an increase in phosphorylation at the T12 residue upon inhibition of the TOR pathway [33]. The fact that both *tor2-287* and *can1-1*/*any1-523C>T* show canavanine resistance but the Any1 PTM pattern is different shows that the mechanism of canavanine resistance differs but, in both cases, involves Any1. Nakashima et al. reported that Any1^K263R^ is sensitive to canavanine but the protein does not exhibit any PTM, which contrast with Any1^R175C^. This can be explained by the fact that the Any1^K263R^ protein is not functional and mimics *any1* deletion. In contrast, Any1^R175C^ is resistant to canavanine and the protein shows a strong decrease in PTMs; hence the impact of the unknown PTM cannot be excluded.

In humans, the Tuberous Sclerosis Complex is a pathology caused by mutations in the TSC1-TSC2 complex. Mutations in one of the tumor suppressor genes, *TSC1* or *TSC2*, lead to an aberrant hyperactivation of the mammalian TOR pathway. Yet, treatments of TSC with mTOR inhibitors are not always efficient [34]. In light of our results, it is conceivable that the defects observed in *TSC1* or *TSC2* deficient cells may involve an arrestin-mediated mechanism.

## Supporting information

S1 Fig A point mutation in *any1* is responsible for canavanine resistance in the *can1-1* strain (related to Fig 2). Serial 10-fold dilutions of indicated strains spotted on YES or synthetic medium lacking arginine and containing the indicated amount of ammonium chloride and canavanine (Can). Pictures taken after 4 and 8 days of incubation at 30⁰C. *any1-523C>T* grows slightly slower than can1-1 on canavanine-containing plates in the presence of low amounts of ammonium chloride (0.5 and 5 g/L).
(TIF)

S2 Fig Expression of Any1-R175C leads to Cat1 internalization (related to Fig 5). **A.** Cellular localization of Cat1-GFP in WT and *can1-1*. Left panel: cells expressing Cat1-GFP observed under a fluorescent microscope. Right panel: intensity of GFP signal across 4 different cells (numbered from 1 to 4 on the left panel) for each genotype. Using Image J, three lines were drawn across the top of the indicated cell and the profile plot was generated for each line. In contrast to *can1-1* cells, the general pattern for WT cells is the presence of two distinct peaks indicating an accumulation of the GFP signal at the cell surface. **B.** Relative intensity of GFP signal between the cell surface and the cytoplasm in indicated strains. GFP intensity was measured by drawing a line at the cell surface (black line on the left panel) and three lines in the cytoplasm (red lines in the left panel) because of the non-homogeneity of the GFP signal in the cytoplasm. The relative intensity of GFP signal was calculated by dividing the intensity at the cell surface by the average intensity in the cytoplasm. Data are shown as the median ± 95% CI. The ratio in WT and *any1*Δ is higher than 1, which suggests that Cat1-GFP molecules are more concentrated at the cell surface than the cytoplasm. The ratio in WT and *any1*Δ is higher than in *any1-523C>T* and *can1-1*, which is consistent with the fact that Cat1-GFP is more internalized in the latter strains. These results correlate with the canavanine sensitivity of WT and *any1*Δ strains and canavanine resistance of *any1-523C>T* and *can1-1* strains.
(TIF)

S1 Raw images (PDF)
