# Supplementary figures and images for: Characterization of canavanine-resistance of cat1 and vhc1 deletions and a dominant any1 mutation in fission yeast
Source: PLoS One. 2022 May 31;17(5):e0269276. doi: 10.1371/journal.pone.0269276 (PMC9154178; doi:10.1371/journal.pone.0269276)

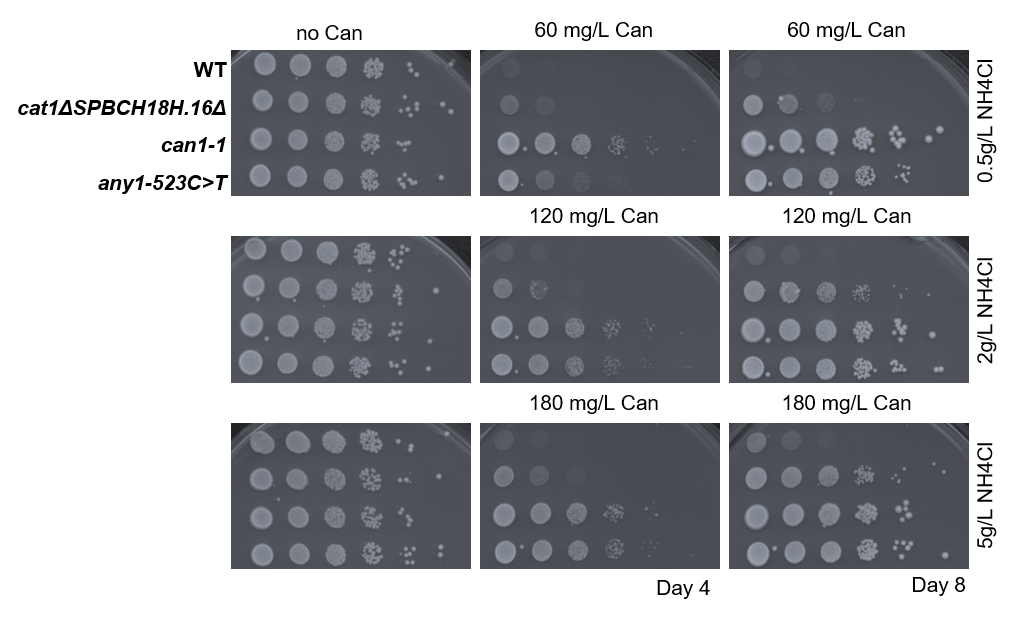

Supplement: S1 Fig — Serial 10-fold dilutions of indicated strains spotted on YES or synthetic medium lacking arginine and containing the indicated amount of ammonium chloride and canavanine (Can). Pictures taken after 4 and 8 days of incubation at 30⁰C. any1-523C>T grows slightly slower than can1-1 on canavanine-containing plates in the presence of low amounts of ammonium chloride (0.5 and 5 g/L). (TIF) [file pone.0269276.s001.tif]

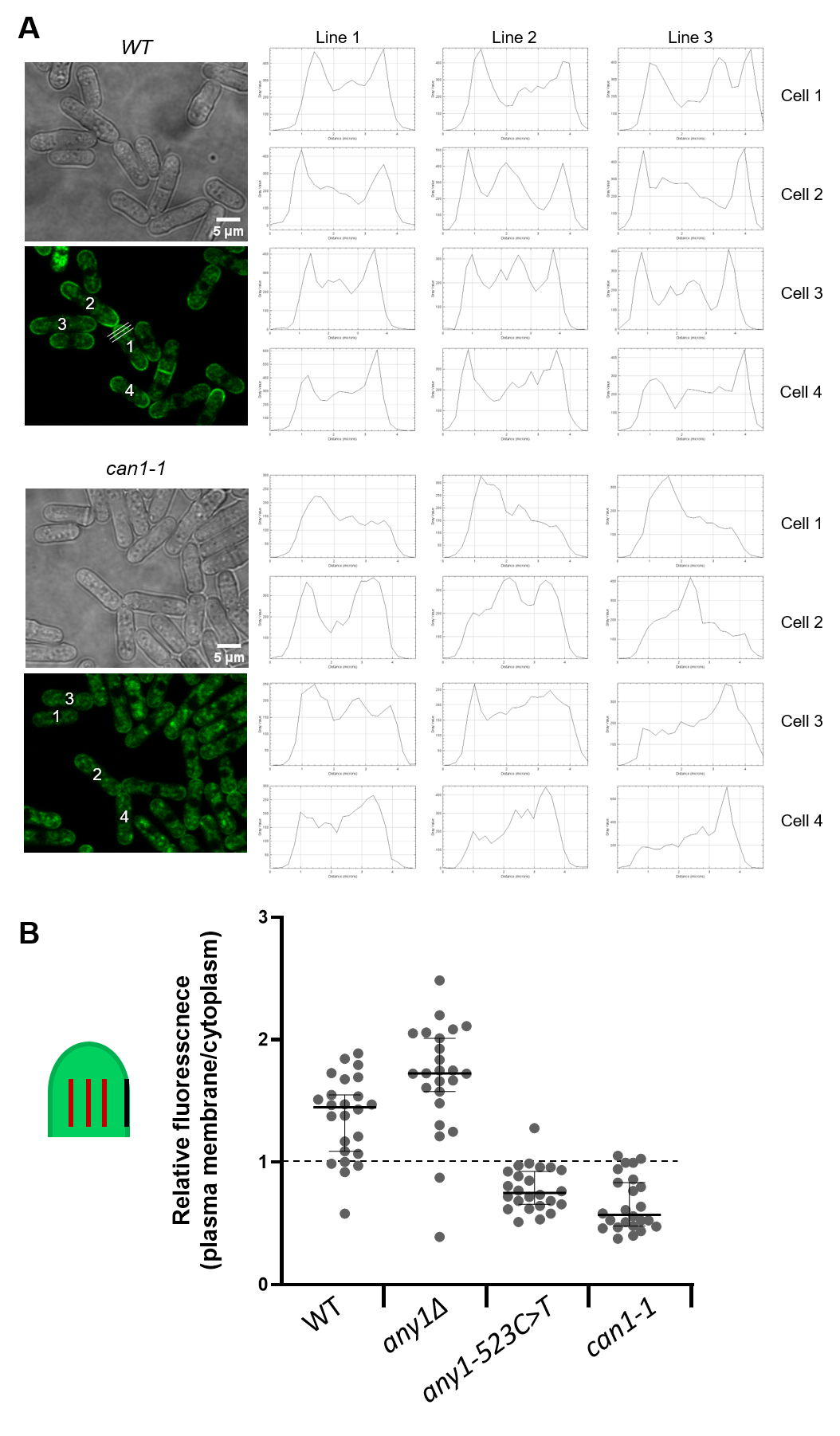

Supplement: S2 Fig — A. Cellular localization of Cat1-GFP in WT and can1-1. Left panel: cells expressing Cat1-GFP observed under a fluorescent microscope. Right panel: intensity of GFP signal across 4 different cells (numbered from 1 to 4 on the left panel) for each genotype. Using Image J, three lines were drawn across the top of the indicated cell and the profile plot was generated for each line. In contrast to can1-1 cells, the general pattern for WT cells is the presence of two distinct peaks indicating an accumulation of the GFP signal at the cell surface. B. Relative intensity of GFP signal between the cell surface and the cytoplasm in indicated strains. GFP intensity was measured by drawing a line at the cell surface (black line on the left panel) and three lines in the cytoplasm (red lines in the left panel) because of the non-homogeneity of the GFP signal in the cytoplasm. The relative intensity of GFP signal was calculated by dividing the intensity at the cell surface by the average intensity in the cytoplasm. Data are shown as the median ± 95% CI. The ratio in WT and any1Δ is higher than 1, which suggests that Cat1-GFP molecules are more concentrated at the cell surface than the cytoplasm. The ratio in WT and any1Δ is higher than in any1-523C>T and can1-1, which is consistent with the fact that Cat1-GFP is more internalized in the latter strains. These results correlate with the canavanine sensitivity of WT and any1Δ strains and canavanine resistance of any1-523C>T and can1-1 strains. (TIF) [file pone.0269276.s002.tif]

Uncropped/Unadjusted image for Figure 4

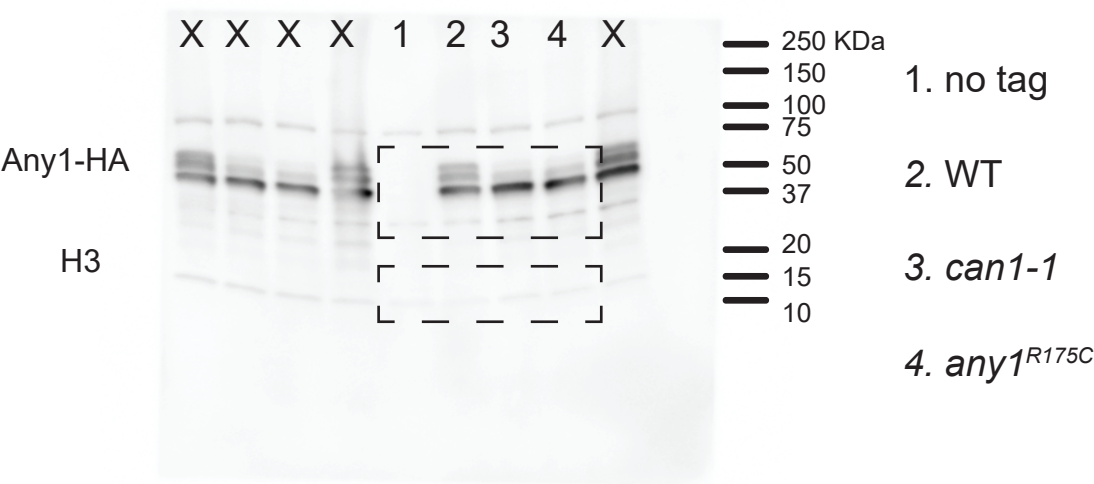

Uncropped/Unadjusted image for Figure 6

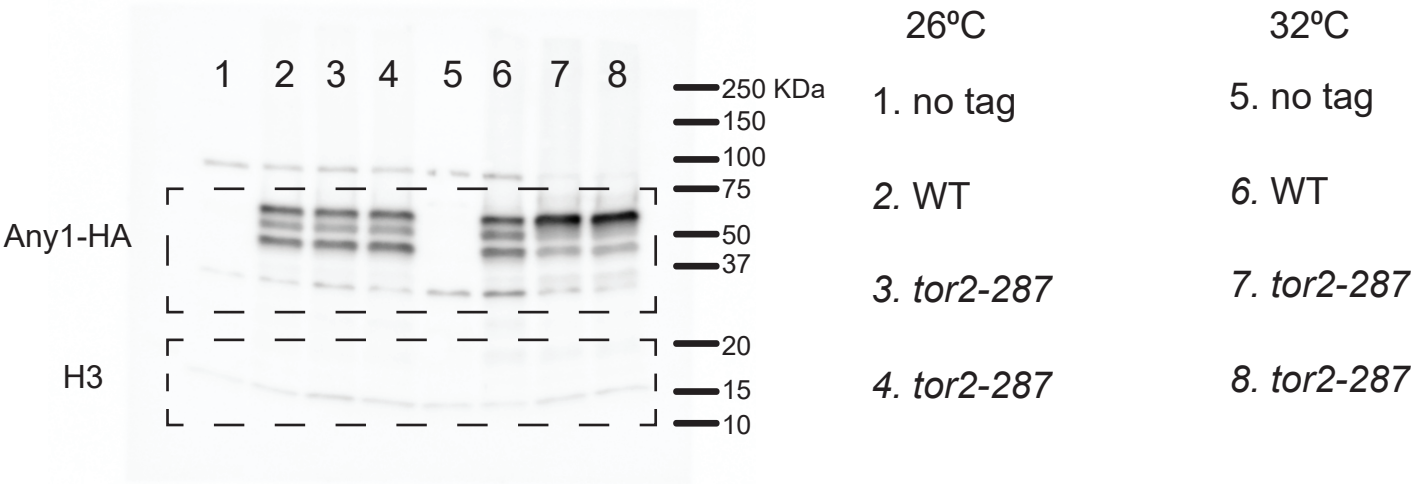

Supplement: S1 Raw images — (PDF) [file pone.0269276.s003.pdf]
